# Revealing the Amylase Interactome in Whole Saliva Using Proteomic Approaches

**DOI:** 10.1155/2018/6346954

**Published:** 2018-01-31

**Authors:** Karla Tonelli Bicalho Crosara, David Zuanazzi, Eduardo Buozi Moffa, Yizhi Xiao, Maria Aparecida de Andrade Moreira Machado, Walter Luiz Siqueira

**Affiliations:** ^1^Schulich Dentistry and Department of Biochemistry, Schulich School of Medicine & Dentistry, The University of Western Ontario, London, ON, Canada; ^2^Department of Pediatric Dentistry, Orthodontics and Public Health, Bauru Dental School, University of Sao Paulo, Bauru, SP, Brazil

## Abstract

Understanding proteins present in saliva and their function when isolated is not enough to describe their real role in the mouth. Due to protein-protein interactions, structural changes may occur in macromolecules leading to functional modulation or modification. Besides amylase's function in carbohydrate breakdown, amylase can delay proteolytic degradation of protein partners (e.g., histatin 1) when complexed. Due to its biochemical characteristics and high abundance in saliva, amylase probably interacts with several proteins acting as a biological carrier. This study focused on identifying interactions between amylase and other proteins found in whole saliva (WS) using proteomic approaches. Affinity chromatography was used, followed by gel electrophoresis methods, sodium dodecyl sulfate and native, tryptic in-solution and in-gel digestion, and mass spectrometry. We identified 66 proteins that interact with amylase in WS. Characterization of the identified proteins suggests that acidic (pI < 6.8) and low molecular weight (MW < 56 kDa) proteins have preference during amylase complex formation. Most of the identified proteins present biological functions related to host protection. A new protein-amylase network was constructed using the STRING database. Further studies are necessary to investigate individualities of the identified amylase interactors. These observations open avenues for more comprehensive studies on not yet fully characterized biological function of amylase.

## 1. Introduction

Whole saliva (WS) is a complex solution that results from secretions from major and minor salivary glands, oral mucosa cells, microorganisms, and elements from the plasma, which reach saliva via gingival crevicular fluid [1]. WS participates in different mechanisms related to the processing of food, the protection of hard and soft oral tissue, and the oral microorganisms' homeostasis [2]. In fact, most of the functions attributed to WS are executed by the salivary proteins [2]. An example of the functionality of salivary proteins is the formation of the Acquire Enamel Pellicle (AEP), a protein layer formed mainly by salivary proteins with higher affinity for hydroxyapatite [3]. Primarily, the AEP works as a physical and chemical barrier that protects the teeth. However, oral microorganisms also use the AEP as a platform to selectively adhere to the tooth surface leading to the formation of the oral biofilm (dental plaque) [3–8].

The presence of the oral biofilm is determinant for the development of the two most prevalent oral diseases: dental caries and periodontal disease. These diseases are the result of an unbalanced situation regarding the host's ability, in part provided by the salivary proteins, to control the growth of pathogenic oral bacteria when compared with the presence of indigenous microorganisms [9].

Several salivary proteins have been explored as key factor for the development of oral diseases based on biofilm formation [8, 10–16]. For example, carbonic anhydrase VI has been investigated as a potential modulator for dental caries progression [14, 15]. This protein is involved in the maintenance of the salivary physiological pH, by the bicarbonate buffer system, and in the neutralization of acid produced by cariogenic microorganisms present in the biofilm [14, 17]. It has been suggested that reduced abundance or activity of carbonic anhydrase VI could be associated with higher risk to develop dental caries [15, 18, 19].

Salivary amylase is another protein with potential correlation with oral diseases. Amylase is the most abundant protein found in human saliva. Amylase is also present in the secretion of mammary and lacrimal glands [20]. Despite the vast literature on salivary amylase, the main function of salivary amylase as an efficient initiator of food digestion in the oral cavity is still debatable [21, 22]. Mechanisms that associate salivary amylase with the clearance of microorganisms from the oral cavity [22–24] and participation in the formation of the AEP [3, 22] and in the modulation of the oral biofilm via bacteria adhesion [8, 23–25] are well explained if considering the protein isolated. However, studies on the salivary proteome have indicated that understanding the individual proteins present in saliva, as well as how they function when isolated, is not enough to describe their real role when in the oral cavity. In fact, most proteins interact with other proteins originating protein complexes. Such interactions may cause structural changes in the macromolecule leading to the modulation or modification of the original individual function of the protein. For instance, when the* in vivo* identified amylase-histatin 1 complex was tested* in vitro*, amylase maintained its enzymatic activity on the hydrolysis of starch, while histatin 1 showed reduced killing activity against* Candida albicans [26]*. Also, it was shown that the lifetime of histatin 1, when complexed with amylase, was significantly increased when exposed to WS [26]. The observation that amylase can delay the proteolytic degradation of salivary protein partners when complexed suggests that this salivary protein may behave as an ideal carrier for important proteins throughout the oral cavity, while maintaining their integrity [26–28].

Heterotypic complexes in saliva between amylase and MUC 5B [28], MUC 7 [29], histatin 1 [26], and histatin 5 [27] have been previously described. Due to the biochemical characteristics and abundance of amylase in saliva, it is very likely that amylase interacts with several other proteins forming complexes. The objective of our study was to reveal the interactions among amylase and other salivary proteins in WS. A comprehensive identification of* in vivo* salivary amylase complexes opens new avenues for further studies related to potential protein degradation stability and how these physiological complexes can be translated to an emerging area related to protein/peptide protection and delivery in a target area.

## 2. Materials and Methods

### 2.1. Ethics Approval for Human Participants

This research was approved by the Research Human Ethics Board of the University of Western Ontario (review number 16181E).

### 2.2. Collection of Whole Saliva (WS) and Preparation of Sample Pools

**Figure 1:**
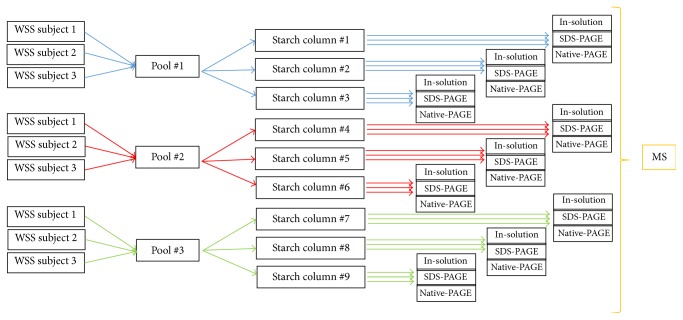
Schematic representation of the adopted methodology.

Stimulated saliva samples were collected from three healthy, nonsmoking adult volunteers, ranging in age from 38 to 42 years (one male and two females). All volunteers exhibited good oral health and overall good systemic conditions. The collection of WS was done between 10:00 AM and 11:00 AM, to reduce the effects of the circadian cycle. Volunteers chewed on a 5 × 5 cm piece of parafilm until 7 mL of saliva was reached. Centrifugation at 14000 ×g for 20 min at 4°C was used to separate pellet and the WS supernatant (WSS). Only WSS were pooled together. Pellets were discarded. Each pool was made with 5 mL of WSS from each volunteer. Three pools were prepared, in different dates. Detailed scheme is shown in Figure 1. Saliva was used fresh for all experiments and was kept on ice from collection to the preparation of aliquots [30]. No protease inhibitors were added to the saliva samples.

### 2.3. Separation of Amylase Complex from WSS Using an In-House Affinity Chromatography

Affinity Chromatography (AC) was employed to enrich amylase when complexed with its protein partners. Potato starch (Acros Organics, New Jersey, USA) was used as ligand and amylase as a target. The used in-house AC method was designed and optimized, inspired by previous study [31]. A sample of 1 mL of pooled WSS was submitted to the column containing 700 *μ*g of starch and hand-pressed slowly, the column was washed with distilled water, and amylase and its complex partners enriched solution was eluded with 1 mL of 0.1% trifluoroacetic acid (TFA). The eluate was subjected to bicinchoninic acid assay (BCA) (Pierce Chemical, Rockford, USA) for total protein concentration measurement. Bovine serum albumin was used as protein standard. Aliquots of 20 *μ*g protein were prepared and subjected to further separation and characterization. Following the enrichment with AC, the amylase-enriched samples were subjected to three distinctive methods: (1) in-solution tryptic digestion, (2) further separation in SDS-PAGE and in-gel tryptic digestion, and (3) confirmation of the complex formation by molecular mobility in the native-PAGE and in-gel tryptic digestion of the amylase complex.

### 2.4. In-Solution Digestion

Aliquots of 20 *μ*g of total protein each were denatured and reduced by addition of 50 *μ*L of 4 M urea, 10 mM DTT, and 50 mM NH_4_ HCO_3_, pH 7.8, and incubated for 1 hour at room temperature (RT). The solution was diluted with the addition of 150 *μ*L of 50 mM NH_4_ HCO_3_, pH 7.8. After tryptic digestion, carried out for at least 16 hours, at 37°C, with 2% w/w sequencing-grade trypsin (Promega, Madison, WI, USA), samples were desalted (Zip Tip C-18, EMD Millipore Inc., Germany) and submitted to mass spectrometric analysis (LC-ESI-MS/MS).

### 2.5. Sodium Dodecyl Sulfate Polyacrylamide Gel Electrophoresis (SDS-PAGE) Separation Followed by In-Gel Digestion

**Figure 2:**
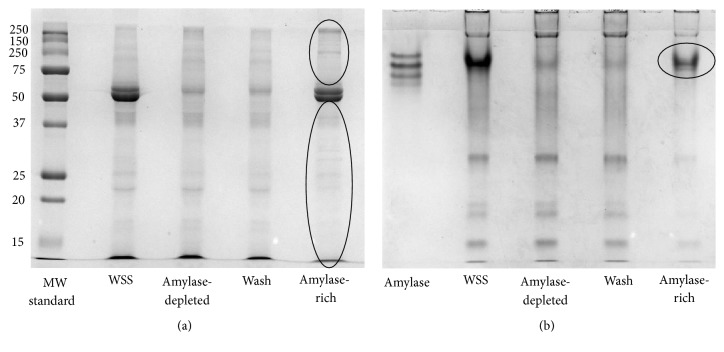
(a) SDS-PAGE 12% and (b) native-PAGE 8% showing areas of interest for identification of protein partners of amylase. The potential protein partners of amylase are expected to be found in the areas marked with an oval shape.

SDS-PAGE was used to separate our protein mixture based on the individual molecular weight (MW) of our proteins. Before loading in the 12% SDS-PAGE, all samples were resuspended in 20 *μ*L of sample buffer (0.4 M Tris-HCl pH 6.8, 4% SDS, 10% glycerol, 0.4% bromophenol blue, and 2% 2-mercaptoethanol) and boiled for 5 min. Each sample was loaded in a separate well. From left to right, the first well was loaded with 5 *μ*L of protein standard (Precision Plus Protein™ All Blue Prestained Protein Standards, Bio-Rad, California, USA), the second well was loaded with a sample from our original solution (WSS), the third well showed the amylase-depleted saliva, the fourth well had sample from the wash of the column, and the last well was loaded with an aliquot containing our amylase-enriched solution (amylase recovered from the starch column along with its partners) (Figure 2(a)). The voltage was kept constant at 100 V during electrophoresis. Immediately after the run, all gels were stained with Coomassie Blue (40% methanol, 10% acetic acid, and 2 g Coomassie Blue) overnight with shaking at RT. Destaining was done the following morning (40% methanol, 10% acetic acid), for 1 hour and 30 min with shaking at RT. After destaining protocol, the gels were kept in Milli-Q water until scanning.

Using a razor blade, regions containing potential partners of amylase were excised from the gels. In the SDS-PAGE, the partners are expected to be found dispersed in the entire lane representing the “amylase-enriched” solution (Figure 2(a)). Each lane was separated into six band regions, and a template was used to ensure that the spots from all gels were extracted at the same MW range. After placement in separate polycarbonate tubes, each band region was cut into approximately 1 × 1 mm pieces. Gel pieces were then destained using 25 mM NH_4_ HCO_3_ in 50% acetonitrile (ACN), shrunk with 100% ACN, and subjected to in-gel tryptic digestion. The digestion was carried out in 25 mM ammonium bicarbonate solution containing 0.01 *μ*g/*μ*L sequencing-grade trypsin (Promega, Madison, WI), for 16 hours at 37°C. Peptide extraction was achieved. Samples were desalted (Zip Tip C-18, EMD Millipore Inc., Germany) before mass spectrometry.

### 2.6. Native-PAGE and In-Gel Digestion

A native gel was used to ensure that amylase would run still complexed with its protein partners. For the 8% native-PAGE, after resuspending the samples of 20 *μ*g of protein with 20 *μ*L of sample buffer (0.4 M Tris-HCl pH 6.8, 10% glycerol, and 0.4% bromophenol blue), the same order used in the SDS-PAGE was observed when loading the samples into the wells from left to right (Figure 2(b)). Native-PAGE running buffer was added to the electrophorese unit, and the voltage was kept constant at 100 V. The same staining method was used with Coomassie Blue overnight as described above. Destaining was done the following morning (40% methanol, 10% acetic acid), for 1 hour with shaking.

For the native-PAGE, the protein partners of amylase are expected to be found in the dark band correspondent to the molecular mobility of the amylase complex (Figure 2(b)). Only the band about the molecular mobility of amylase-protein complex was studied. As described above, gel bands were cut into small 1 × 1 mm pieces, destained, and subjected to in-gel tryptic digestion. The digestion was carried out in the same manner that was described for the SDS-PAGE. Peptides were recovered and samples were desalted (Zip Tip C-18, EMD Millipore Inc., Germany) before mass spectrometry.

### 2.7. MS Analysis

Samples from all three described approaches were resuspended in 97.5% distilled water/2.4% ACN/0.1% formic acid and then subjected to RP nLC-ESI-MS/MS, using a LTQ-Velos (Thermo Scientific, San Jose, CA, USA) mass spectrometer. LC aligned with the C18 column of capillary-fused silica (column length 10 mm, column id 75 m, 3 m spherical beads, and 100 A° pores size) was used, linked to the MS through ESI. The survey scan was set in the range of *m*/*z* values 390–2000 MS/MS. Peptides were eluted from the nanoflow RP-HPLC over a 65 min period, with linear gradient ranging from 5 to 55% of solvent B (97.5% ACN, 0.1% formic acid), at a flow rate of 300 nL/min, with a maximum pressure of 280 bar. The electrospray voltage was 1.8 kV and the temperature of the ion-transfer capillary was 300°C. After a MS survey scan range within *m*/*z* 390–2000 was performed and after selection of the most intense ion (parent ion), MS/MS spectra were achieved via automated sequential selection of the seven peptides with the most intense ion for CID at 35% normalized collision energy, with the dynamic exclusion of the previously selected ions. The MS/MS spectra were matched with human protein databases (Swiss-Prot and TrEMBL, Swiss Institute of Bioinformatics, Geneva, Switzerland, https://ca.expasy.org/sprot/) using SEQUEST algorithm in Proteome Discoverer 1.3 software (Thermo Scientific, USA). The searches were performed by selecting the following SEQUEST parameters: (1) trypsin as protease enzyme, (2) 2 Da precursor ion mass tolerance, (3) 0.8 Da fragment ion mass tolerance, and (4) dynamic modifications of oxidized cysteine and methionine and phosphorylated serine and threonine. A maximum of four dynamic modifications per peptide were accepted. The SEQUEST score filter criteria applied to the MS/MS spectra for peptides were absolute XCorr threshold 0.4, fragment ion cutoff percentage 0.1, and peptide without protein XCorr threshold 1.5. Any nontryptic peptides passing the filter criteria were discarded. Only proteins for which two or more peptides were identified are reported in this study.

### 2.8. Identification of Protein Partners of Amylase

After MS analysis and interpretation, comparison of the common partners among the used methods allowed the construction of a list with proteins that participate with amylase in salivary complexes. Three in-house AC columns (technical triplicate) were used for each one of the 3 saliva pools (biological triplicate) prepared in different dates, making a total of 9 replicates for each one of the used approaches (in-solution digestion, SDS-PAGE followed by in-gel digestion, and native-PAGE followed by in-gel digestion). For the approach using in-solution digestion, the proteins identified by MS for the 9 replicates were compared, and proteins that were identified in at least 2 of the replicates were listed as common proteins for this first approach. Similarly, the proteins identified by MS from the dark bands of the 9 replicates submitted to native-PAGE, followed by in-gel digestion, were compared and the proteins that appeared in at least two replicates were considered common protein partners for this second method. Last, to identify the amylase partners from the lines representing the amylase-enriched sample in the 9 replicates submitted to the SDS-PAGE approach, followed by in-gel digestion and MS, a template was used to extract the bands from all the 9 gels at approximately the same molecular weight range. The lanes with enriched sample were divided into 6 areas. Each area was analyzed separately and the 6 protein lists for each line were combined into one single protein list for each replicate; duplicate proteins were excluded. Like the other two approaches, proteins identified in at least two of the 9 replicates were deemed common for this third approach. After this triage, a Venn diagram was used to verify similarities among the common proteins listed from each described approach. Inclusion criterion for positive identification of proteins as complex partners of amylase was that the same protein was found in at least two of the used approaches.

### 2.9. Bioinformatics Characterization of Amylase Complex Partners

The proteins identified in at least two of the described approaches were then characterized based on their calculated isoelectric point (pI) and molecular weight (MW). Using the physiologic salivary pH as reference (pH 6.8), the identified proteins were grouped based on their pI (pI below and above 6.8). In addition, the MW of amylase (56 kDa) was assumed for our MW cut-off and the same proteins were divided in three groups: proteins with 0–20 kDa, proteins with 20–56 kDa, and proteins with MW above 56 kDa. Whenever available, pI and MW were calculated after removal of signal peptide given by UniProt database. Otherwise, pI and MW informed in the MS report were adopted. The identified amylase complex partners were also classified based on their biological functions using data from UniProt (http://uniprot.org) assessed on August 2017. Four major groups were formed including proteins that exhibit antimicrobial activity, protection against chemical aggression, participation in host immune response and/or regulation of inflammation, and physical protection of the oral mucosa and/or wound healing.

### 2.10. Simulation of Amylase-Protein Network Using STRING Database

STRING database was used to provide a schematic representation of the interactions among amylase and other proteins found in the human WS as described elsewhere [32]. First, a comprehensive search was performed in eight different databases (BioGRID, HPRD, APID, EMBL-EBI, FpClass, STRING, IntAct, and BioPlex) (Table 1) to provide a solid list with both known and predicted protein-amylase interactions. Second, a simulated amylase hub containing only the 66 proteins identified in this study was constructed using the STRING database. Last, a more inclusive network was created by merging the hub containing the proteins identified in this study with the possible partners of amylase listed in all eight searched databases. The filter was set to match with human databank, and the confidence score was set to 0.4 (medium) in all representations.

## 3. Results

The selectivity of our in-house AC starch columns towards amylase is demonstrated in Figure 2, where the band related to amylase's MW (56 kDa) in Figure 2(a) and the amylase complex in Figure 2(b) practically disappear in the lines representing saliva depleted from amylase and the wash with distilled water. On the other hand, dark bands are seen in the corresponding areas with the amylase-enriched samples. Although slight bands can be seen in areas besides that of the amylase complex in Figure 2(b) where the enriched sample is represented, such faded bands might be related to proteins that either show weak interaction with the complex which was disrupted during processing of samples, or may be related to “contaminants” that remained in the column after wash. To ensure a precise identification of proteins from the complex, only the proteins listed in the dark band in the native-PAGE (Figure 2(b)) were considered.

**Figure 3:**
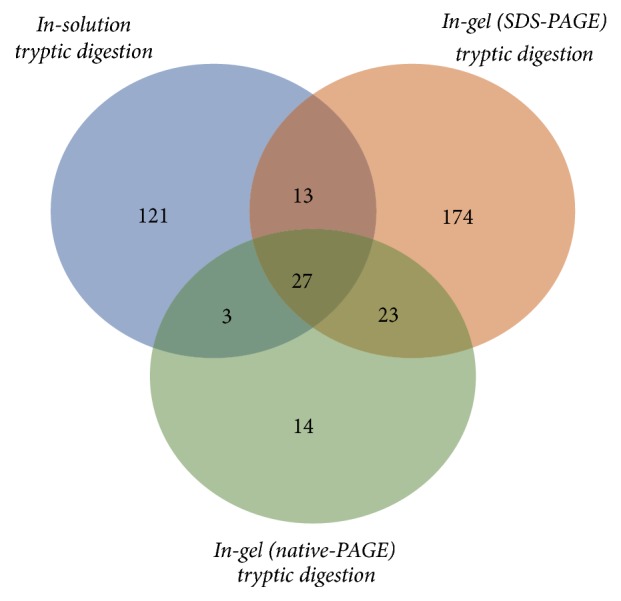
Venn diagram distribution of identified proteins in each of the three proteomic approaches used in this study.

The data obtained after LC-ESI-MS/MS analysis of samples from the three described approaches identified 66 different proteins found in WS that form complex with salivary amylase. All identified proteins are listed in Table 2, along with the corresponding approach used for the identification, protein MW and pI. When results from all approaches were combined, 375 different proteins were recognized. In-solution digestion provided 164 proteins that probably interact with amylase: SDS-PAGE, followed by in-gel tryptic digestion, 237 potential partners; native-PAGE, followed by in-gel digestion, 67 possible complex partners. After selecting only proteins that were identified in two or more of the used approaches, results were narrowed down to 66 proteins, where 27 proteins were detected in all three methods, besides amylase itself, and 39 other proteins were concomitantly identified in only two of the used approaches. A total of 13 unique proteins were identified using both in-solution tryptic digestion and SDS-PAGE, followed by in-gel tryptic digestion; 23 proteins were found in both PAGE approaches; and 3 proteins were uniquely found concomitantly in the samples from in-solution tryptic digestion and native gels, followed by in-gel tryptic digestion (Figure 3).

**Figure 4:**
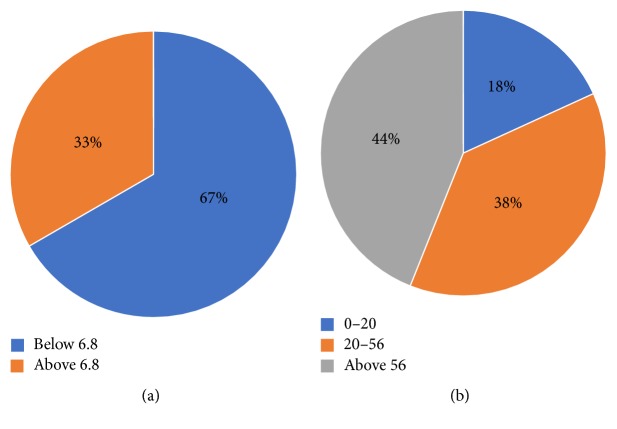
Percentage distribution of the identified proteins according to the biochemical characteristic of salivary amylase (pI 6.8 and 56 KDa). (a) pI distribution of the identified proteins using salivary amylase pI as comparison value. (b) Molecular weight distribution of the identified proteins using salivary amylase molecular weight as comparison value.

Moreover, the 66 common proteins displayed MW ranging from 4.31 kDa to 3340.16 kDa (Table 2), where most of the identified amylase partners (56%) presented a MW below 56 kDa, amylase's MW (Figure 4(a)). The identified proteins were also grouped based on their isoelectric points (pI). Clearly, most of the 66 proteins (67%) presented pI below 6.8. One-third (33%) of the identified amylase-protein partners exhibited basic characteristics ranging in pI above 6.8 (Figure 4(b)).

Interestingly, the characterization of the 66 identified proteins based on their biological functions indicated that most of the proteins participating in complex with amylase exhibit protective roles towards the maintenance of the host's health. In fact, from the 66 identified proteins, 37 display oral defensive functions: 13 proteins have antimicrobial activities, 9 elements are capable of neutralizing chemical aggressions to the host's tissues, 10 proteins participate in mechanisms that initiate or modulate the host's immune response and inflammatory process, and 10 proteins contribute to the physical protection of the host's tissue and/or wound healing (Table 3).

**Figure 5:**
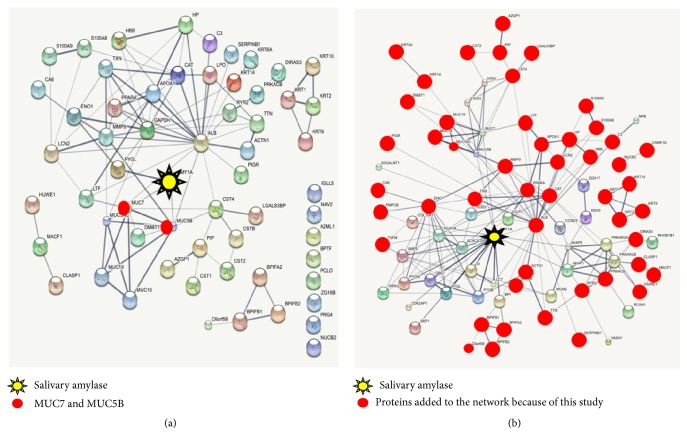
In silico view of amylase interactome using STRING database. (a) All proteins identified herein interact with amylase forming complex where represented. (b) An inclusive amylase interactome was constructed merging the proteins identified in this study with all known (*in vitro* studies) and predicted proteins (in silico databases) previously mentioned to form complex with amylase.

The amylase interactome simulation using STRING database demonstrated that not all 66 proteins were linked to the protein-amylase network (Figure 5(a)). Three distinct isolated groups of 3 to 4 proteins were formed apart from the network, along with other lonely individual nodes. MUC 7 and MUC 5B were among the identified proteins. When the newly identified amylase network was merged with the previously documented interactors, only two groups of proteins were not linked to the network (Figure 5(b)). One group contained NUCB2 (nucleobindin-2) and CAMK1D (calcium/calmodulin-dependent protein kinase type 1D), and the other cluster formed by four keratins.

## 4. Discussion

A total of 66 proteins that participate in protein complex with amylase in WS were identified with the application of three different proteomic approaches. Initially, AC was used to enrich amylase along with its partners from the complex saliva solution. The reaction between amylase and starch is an enzymatic reversible mechanism [20], allowing for the recovery of intact amylase after its reaction with the starch. The use of a starch column was previously described as a mean for depletion of amylase from saliva [31]. However, in this study, we demonstrated that the mentioned method enriches amylase from saliva still complexed with other proteins. This observation was first suggested by the different bands present in the SDS-PAGE when the samples eluted from the starch column were separated by MW (Figure 2(a)). Later, this observation was confirmed by MS analysis of the same amylase-enriched samples where many proteins besides amylase were identified (Table 2). Thus, AC starch column alone should not be recommended for the depletion of amylase from saliva, unless a careful dismemberment of protein complexes can be performed earlier in ways that do not interfere with the activity of salivary amylase.

Moreover, the importance of using different methods for the identification of proteins was here demonstrated. Combining all used approaches, a total of 375 unique proteins were identified as potential members of the amylase complex. Interesting to note, SDS-PAGE was the method where the largest number of proteins was identified (237 proteins). From the 66 proteins that were identified in at least two of the used approaches and therefore are more likely to interact with amylase, only 3 proteins were not identified in the approach with SDS-PAGE. This demonstrates that additional sample separation based on the MW of each protein, together with the MS analysis of independent bands from different areas of the gel, prevents highly abundant proteins from masking or hiding low abundant ones, therefore improving the method specificity. On the other hand, while using directly in-solution tryptic digestion uniquely, 24 proteins from our final list of 66 interactors were not identified (Figure 3), once again reinforcing the hypothesis of high abundance proteins preventing the identification of low abundance ones unless further separation is performed before MS analysis. Also, 13 of the 66 identified proteins from the amylase complex were not identified in the native-PAGE approach. Since native gels provide a sample separation based on the molecular mobility and charge of the complex, the absence of some of the identified proteins may be a consequence of weaker bindings, thus damaging the stability of some protein-protein interactions and preventing all proteins that were originally in the complex from being identified in this method.

Amylase-protein-protein interactions with histatins (histatin 1 and histatin 5) and with mucins (MUC 5B and MUC 7) were described previously [26, 28, 29, 33]. Mucins (MUC5B and MUC7), a protein family only present in mucous glands such as submandibular and sublingual glands, were here identified among the partners of amylase in WS, confirming previous studies [28, 29]. Contrarily, histatins were not identified in this study probably because of their short lifespan in the oral cavity due to protein degradation by endogenous oral proteases [26, 34, 35]. Protease inhibitors can be used in an attempt to prevent proteolytic degradation. However, in saliva, it has been shown that short-term storage of freshly collected saliva samples on ice is more effective in preventing proteolytic degradation, without interfering with the chemistry of the proteome, than the use of protease inhibitors [30]. Therefore, no protease inhibitors were added to the saliva samples as they could promote chemical alterations on our protein complexes leading to changes in the stability of the complex and to an incorrect identification of the proteins that participate in complexes with amylase.

To distinguish a protein profile among the identified partners of amylase, biochemical characterization was performed according to the calculated pI and MW of the proteins and to their biological functions. Using the prevailing physiological salivary pH as reference (pH 6.8), the identified proteins were divided into two groups: pI below and pI above 6.8. Most of all identified proteins (67%) presented isoelectric points below 6.8 and therefore exhibited negative charge in a solution with pH 6.8. On the other hand, one-third of the identified amylase-protein partners (33%) exhibited more basic characteristics with pI above 6.8, showing positive charges in pH 6.8. Therefore, there appears to be a preference for acidic proteins (pI < 6.8) to participate in the identified amylase complex. Knowing that ionic forces and hydrogen bonding, both electrostatic interactions, are involved in the formation of protein complexes, shifts in the net charge of salivary proteins possibly interfere with the nature and abundance of the proteins present in complexes. Differences among the pH of the secretions from the major salivary glands have been described [36, 37]. Also, changes in the pH of saliva have been suggested as biomarkers for systemic diseases [38]. In tumors, for example, there seems to be a shift in pH towards being acidic, acting as a favorable factor for tumor cells [38]. The proposition that variations in the salivary pH might interfere in the formation of salivary complexes suggests a new research and diagnostic avenue combining salivary proteome/interactome and salivary pH.

Since only subjects with overall good systemic and oral health were included in this study, it is implied that all our results were acquired around physiological salivary pH. In this condition, the characterization of the 66 identified proteins based on their biological functions reinforced the possible function of amylase as an important biological carrier. In total, 56% (37 proteins) of the identified partners of amylase exhibited important roles towards the maintenance of oral health. Four main mechanisms were recognized: antimicrobial activities, protection against chemical aggressions, immune response and regulation of inflammation, and physical protection of the mucosa and wound healing. About the debatable participation of amylase in the development of dental caries, this study did not aim to clarify the direct involvement of salivary amylase in the carious process. Contrarily, a new question is here proposed on the potential indirect participation of amylase in the protection against dental caries via functional modulation and/or protection of “anticariogenic” proteins from early proteolytic degradation in the oral cavity. A possible example of such proteins identified in this study is carbonic anhydrase VI. Besides carbonic anhydrase VI's involvement in taste sensation, this isoenzyme maintains the physiological salivary pH by catalyzing the hydration of carbon dioxide (bicarbonate buffer system), assisting in the recovery from acidic, more cariogenic, salivary challenges [17]. Carbonic anhydrase VI can also penetrate in the biofilm to facilitate the neutralization of acids secreted by the bacteria [14]. Carbonic anhydrase VI was identified among the proteins that participate in salivary complex with amylase. However, the direct binding of amylase and carbonic anhydrase VI and the possible consequences of such interaction are yet to be investigated. Other proteins identified in this study were cystatins B, SN, S, and SA. Cystatins are proteins that inhibit cysteine proteases secreted by the host, bacteria, and viruses [39]. Cystatins SA and SN are particularly involved in the control of the proteolytic events* in vivo* such as periodontal tissue destruction [40]. The presence of cystatins B, SN, S, and SA in salivary complexes with amylase suggests that amylase may contribute indirectly against periodontal diseases.

Open proteomics/interactomics databases have been developed to assist in the study of protein-protein interactions and to accelerate discoveries in the field. Using the STRING database, a simulation of the amylase interactome with the identified partners of amylase was constructed. Out of the 66 members of the amylase complex listed in this study, only two proteins had been previously reported in the literature to present direct interactions with amylase; they were MUC5B [28] and MUC7 [29]. No direct binding between amylase and any of the other 64 proteins identified herein has been described up till now. Therefore, additional studies are needed to determine if any of these other proteins bind directly to amylase forming the first shell of the protein complex, as well as the exact position of each of the identified members of the amylase complex in the protein-protein network. Furthermore, the creation of a second amylase-protein-protein network merging the newly identified amylase-protein network with the previous known and predicted amylase interactors demonstrated that, using in silico approach based on molecular affinity prediction and prior* in vivo* and* in vitro* experiments, most the 66 proteins identified herein fill the gap in the amylase interactome present in WS.

It is important to highlight that the proteins identified herein in complexes with amylase, the most abundant salivary protein, were detected using three different proteomic approaches, with nine replicates, using saliva from three subjects, collected in three different dates (Supplemental Table 1). On the other hand, it is likely that each salivary protein has a different binding affinity with amylase. In fact, it is well known that changes in the salivary flow rate, person's overall health, and emotional state can promote qualitative and quantitative variations in the salivary proteome [41–45] and, consequently, in the amylase interactome. Future studies need to address the amylase interactome in different physiological/pathological conditions.

In summary, this study pioneered the exploration of the vast salivary interactome. It is important to remember that some of the proteins identified herein may interact with amylase indirectly, having one or more proteins as mediators of such interactions. Unfortunately, very little is known about the dynamics of these interactions. Transient protein complexes are less likely to be identified than permanent protein complexes. Additional studies are needed to confirm how the proteins listed in this manuscript interact with each other and with amylase. Amylase's ability to protect such partners from proteolytic degradation and/or modulate their biological functions while in the complex is yet to be studied comprehensively.

## 5. Conclusion

The large number of amylase complex partners identified herein reinforces the hypothesis that the real role of amylase in the oral cavity might not be related to carbohydrate digestion. Instead, amylase's most important role may be associated with protein transport and possible protection and functional modulation of its partners. In an era of more personalized and targeted medicine, this study opens the hypothesis for a novel therapeutic avenue where amylase can offer information for the development of an ideal carrier for functionally important peptides/proteins towards the prevention of oral diseases. Moreover, the salivary interactome may function as a foundation for the development of more efficient artificial saliva and/or mouth washes and provide more reliable models to design drugs directed to amylase or dependent on its function.

## Figures and Tables

**Table 1:** List of proteins with known and predicted interactions with amylase, identified by search in eight databases (BioGrid, HPRD, APID, EMBL-EBI, FpClass, STRING, IntAct, and BioPlex).

Protein name	Database
Sucrase-isomaltase (alpha-glucosidase)	STRING
Amylo-alpha-1,6-glucosidase,4-alpha-glucanotransferase	STRING
Lactase	STRING
Bactericidal/permeability-increasing protein	STRING
Collagen, type X, alpha 1	STRING
Glycogen phosphorylase, muscle form	STRING
Acetyl-CoA carboxylase beta (2)	STRING
Uridine monophosphate synthetase	STRING
Acetyl-CoA carboxylase alpha	STRING
S-phase kinase-associated protein 1	STRING
Glucan (1,4-alpha-), branching enzyme 1	STRING
Glycogen phosphorylase, liver form	STRING
Glycogen phosphorylase, brain form	STRING
A kinase (PRKA) anchor protein 8	HPRD, BioGRID, APID, EMBL-EBI, FpClass, STRING
Cyclin-dependent kinase 2 associated protein 1	BioGRID, String
A kinase (PRKA) anchor protein 1	HPRD, BioGRID, APID, EMBL-EBI, STRING
Mucin 5B, oligomeric mucus/gel-forming	BioGRID, APID, EMBL-EBI
Ras association (RalGDS/AF-6) domain family member 6	HPRD, BioGRID, APID, EMBL-EBI
Putative oral cancer suppressor, deleted in oral cancer 1	IntAct, HPRD, APID, EMBL-EBI
Superoxide dismutase (Mn), mitochondrial	IntAct, BioGRID, APID, EMBL-EBI
Uncoupling protein 2 (mitochondrial, proton carrier)	IntAct, APID, EMBL-EBI
ARP8 actin-related protein 8 homolog (yeast)	BioGRID, BioPlex, APID, EMBL-EBI
beta-1,3-N-Acetylgalactosaminyltransferase 1 (globoside blood group)	BioGRID, BioPlex, APID, EMBL-EBI
General transcription factor IIB	BioGRID, BioPlex, APID, EMBL-EBI
Killer cell immunoglobulin-like receptor, two domains, short cytoplasmatic tail, 2	BioGRID, BioPlex, APID, EMBL-EBI
Mab-21-like 1 (*C. elegans*)	BioGRID, BioPlex, APID, EMBL-EBI
Starch binding domain 1	BioGRID, BioPlex, APID, EMBL-EBI
Protein kinase, cAMP-dependent, regulatory subunit, type II, beta	BioGRID, BioPlex, APID, EMBL-EBI
Peptide (mitochondrial processing) beta	BioGRID, BioPlex, APID, EMBL-EBI
Trafficking protein particle complex 12	BioGRID, BioPlex, APID, EMBL-EBI
Ubiquitin-like 7	BioGRID, BioPlex, APID, EMBL-EBI
Calcium/calmodulin-dependent protein kinase ID	BioGRID, BioPlex, APID, EMBL-EBI
Receptor-interacting serine-theorine kinase 3	BioGRID, BioPlex, APID, EMBL-EBI
Chemokine (C-C motif) receptor-like 2	BioGRID, BioPlex, APID, EMBL-EBI
Pleckstrin homology domain containing, family G (with RhoGef domain) member 6	BioPlex, APID
Regulator of calcineurin 1	BioPlex, APID
Vasohibin 1	BioPlex, APID
Gastrokine 1	BioPlex, APID
Zinc-finger, B-box domain containing	BioPlex, APID
DEAD (Asp-Glu-Ala-Asp) box helicase 17	BioPlex, APID
Rho-related BTB domain containing 1	BioPlex, APID
Ts translation elongation factor, mitochondrial	BioPlex, APID
Tumor necrosis factor receptor superfamily, member 19	BioPlex, APID
Neuropeptide B	BioPlex, APID
Forkhead box N4	BioPlex, APID
FERM domain containing 1	BioPlex, APID
WD repeat domain 6	BioPlex, APID
DNA replication licensing factor MCM2	FpClass
Probable ATP-dependent RNA helicase DDX5	FpClass
G1/S-specific cyclin-D3	FpClass
cAMP-dependent protein kinase type II-alpha regulatory subunit	FpClass
Histatin 1	FpClass
Salivary acidic proline-rich phosphoprotein 1/2	FpClass
Statherin	FpClass

Websites: STRING: https://www.string-db.org; HPRD: http://www.hprd.org; BioGRID: https://www.thebiogrid.org; APID: http://apid.dep.usal.es; EMBL-EBI: https://www.ebi.ac.uk; FpClass: http://dcv.uhnres.utoronto.ca/FPCLASS/ppis/; IntAct: https://www.ebi.ac.uk/intact; BioPlex: http://bioplex.hms.harvard.edu.

**Table 2:** List of all identified potential amylase protein partners according to the used proteomic approach.

Accession number	Protein name	In-solution	In-gel (SDS-PAGE)	In-gel (native- PAGE)	MW (KDa)	Calc. pI
C0JYZ2	Titin	x	x	x	3340.16	6.09
B4E1M1	cDNA FLJ60391, highly similar to lactoperoxidase	x	x	x	73.88	8.15
Q9HC84	Mucin-5B	x	x	x	593.84	6.20
P04080	Cystatin-B	x	x	x	11.14	6.96
B4DVQ0	cDNA FLJ58286, highly similar to actin, cytoplasmic 2	x	x	x	37.30	5.71
P01037	Cystatin-SN	x	x	x	14.32	6.92
Q6PJF2	IGK@ protein	x	x	x	23.32	6.98
Q0QET7	Glyceraldehyde-3-phosphate dehydrogenase (fragment)	x	x	x	24.60	8.51
A0A075B6K9	Ig lambda-2 chain C regions (fragment)	x	x	x	11.30	7.24
P05109	Protein S100-A8	x	x	x	10.70	6.57
P12273	Prolactin-inducible protein	x	x	x	13.52	5.40
Q96DR5	BPI fold-containing family A member 2	x	x	x	25.05	5.19
A0A0C4DGN4	Zymogen granule protein 16 homolog B	x	x	x	17.21	5.39
Q9UGM3	Deleted in malignant brain tumors 1 protein	x	x	x	258.66	5.19
P01833	Polymeric immunoglobulin receptor	x	x	x	81.35	5.59
P01876	Ig alpha-1 chain C region	x	x	x	37.66	6.51
P23280	Carbonic anhydrase VI	x	x	x	33.57	6.41
C8C504	beta-Globin	x	x	x	15.87	7.98
A7Y9J9	Mucin 5AC, oligomeric mucus/gel-forming	x	x	x	645.90	6.27
P01834	Ig kappa chain C region	x	x	x	11.60	5.87
H6VRF8	Keratin 1	x	x	x	66.00	8.12
P13645	Keratin, type I cytoskeletal 10	x	x	x	58.83	5.13
P01036	Cystatin-S	x	x	x	14.19	4.83
B2R4M6	Protein S100	x	x	x	4.31	4.55
P35908	Keratin, type II cytoskeletal 2 epidermal	x	x	x	65.43	8.07
B1APF8	cAMP-dependent protein kinase catalytic subunit beta (fragment)	x	x	x	20.56	9.56
B5ME49	Mucin-16	x	x	x	1519.17	5.13
P25311	Zinc-alpha-2-glycoprotein	x	x		32.14	5.58
F6KPG5	Albumin (fragment)	x	x		66.49	6.04
B2R7Z6	cDNA, FLJ93674	x	x		50.34	7.05
E9PKG6	Nucleobindin-2	x	x		37.50	5.01
P02647	Apolipoprotein A-I	x	x		28.08	5.27
Q9Y6V0	Protein piccolo	x	x		553.28	6.09
P09228	Cystatin-SA	x	x		14.35	4.85
A0A024R9Y3	HECT, UBA, and WWE domain containing 1, isoform CRA_a	x	x		479.90	5.21
E7ETD6	Nucleosome-remodeling factor subunit BPTF	x	x		307.90	6.04
Q8TAX7	Mucin-7	x	x		36.81	9.30
P06733	alpha-Enolase	x	x		47.04	6.99
P10599	Thioredoxin	x	x		11.61	4.82
Q9UPN3	Microtubule-actin cross-linking factor 1, isoforms 1/2/3/5	x	x		838.31	5.28
O95661	GTP-binding protein Di-Ras3	x		x	25.50	9.46
A7E2D6	NAV2 protein	x		x	261.56	8.98
G3CIG0	MUC19 variant 12	x		x	802.68	4.96
Q8N4F0	BPI fold-containing family B member 2		x	x	47.13	8.48
P01024	Complement C3		x	x	184.95	6.00
H7BY35	Ryanodine receptor 2		x	x	562.25	6.19
Q07869	Peroxisome proliferator-activated receptor alpha		x	x	52.23	5.86
B4E1T1	cDNA FLJ54081, highly similar to Keratin, type II cytoskeletal 5		x	x	58.81	5.97
A8K2U0	alpha-2-Macroglobulin-like protein 1		x	x	159.33	5.50
Q6P5S2	Protein LEG1 homolog		x	x	35.86	5.79
B4E3A8	cDNA FLJ53963, highly similar to leukocyte elastase inhibitor		x	x	38.69	6.22
F8WA11	CLIP-associating protein 1		x	x	162.66	8.72
B7ZAL5	cDNA, FLJ79229, highly similar to lactotransferrin		x	x	73.10	7.78
P02533	Keratin, type I cytoskeletal 14		x	x	51.56	5.09
B2R825	alpha-1,4-Glucan phosphorylase		x	x	97.01	7.30
A0A087WWT3	Serum albumin		x	x	43.03	5.69
B7Z759	cDNA FLJ61672, highly similar to proteoglycan-4 (fragment)		x	x	92.09	9.44
J3QLC9	Haptoglobin (fragment)		x	x	39.03	5.54
P01877	Ig alpha-2 chain C region		x	x	36.50	6.10
A8K739	cDNA FLJ77339		x	x	24.84	5.06
B7Z747	cDNA FLJ51120, highly similar to matrix metalloproteinase-9		x	x	64.09	6.42
B7Z565	cDNA FLJ54739, highly similar to alpha-actinin-1		x	x	94.72	5.69
B4DI70	cDNA FLJ53509, highly similar to galectin-3-binding protein		x	x	44.37	5.03
P35527	Keratin, type I cytoskeletal 9		x	x	62.06	5.14
P80188	Neutrophil gelatinase-associated lipocalin		x	x	20.55	9.02
P04040	Catalase		x	x	59.62	6.95

**Table 3:** Distribution of proteins identified to interact with salivary amylase forming complex based on their biological functions.

Biological function	Accession number	Protein name
Defense response to bacterium, virus, and fungus (*n* = 13)	Q9HC84	Mucin-5B
	B4E1M1	cDNA FLJ60391, highly similar to lactoperoxidase
	P05109^*∗*^	Protein S100-A8
	Q96DR5	BPI fold-containing family A member 2
	Q8N4F0	BPI fold-containing family B member 2
	Q9UGM3	Deleted in malignant brain tumors 1 protein
	P01876	Ig alpha-1 chain C region
	P01834^*∗*^	Ig kappa chain C region
	P01877^*∗*^	Ig alpha-2 chain C region
	B7Z759	cDNA FLJ61672, highly similar to proteoglycan-4 (fragment)
	Q8TAX7^*∗*^	Mucin-7
	B7ZAL5	cDNA, FLJ79229, highly similar to lactotransferrin
	B4DI70	cDNA FLJ53509, highly similar to galectin-3-binding protein

Neutralization of chemical aggression (*n* = 9)	P04080	Cystatin-B
	P01037	Cystatin-SN
	P23280	Carbonic anhydrase VI
	P01036	Cystatin-S
	P09228	Cystatin-SA
	A8K2U0	alpha-2-Macroglobulin-like protein 1
	B4E3A8	cDNA FLJ53963, highly similar to leukocyte elastase inhibitor
	P04040	Catalase
	A7Y9J9^*∗*^	Mucin 5AC, oligomeric mucus/gel-forming

Immune response and regulation of inflammation (*n* = 10)	P05109^*∗*^	Protein S100-A8
	P12273	Prolactin-inducible protein
	P01834^*∗*^	Ig kappa chain C region
	B2R4M6	Protein S100
	P01024	Complement C3
	P80188	Neutrophil gelatinase-associated lipocalin
	P01877^*∗*^	Ig alpha-2 chain C region
	A0A075B6K9	Ig lambda-2 chain C regions (fragment)
	B7Z747	cDNA FLJ51120, highly similar to matrix metalloproteinase-9
	Q6PJF2	IGK@ protein

Mucosa protection and wound healing (*n* = 10)	B5ME49	Mucin-16
	P01833	Polymeric immunoglobulin receptor
	P35908	Keratin, type II cytoskeletal 2 epidermal
	P25311	Zinc-alpha-2-glycoprotein
	Q07869	Peroxisome proliferator-activated receptor alpha
	Q9UPN3	Microtubule-actin cross-linking factor 1, isoforms 1/2/3/5
	A7Y9J9^*∗*^	Mucin 5AC, oligomeric mucus/gel-forming
	Q8TAX7^*∗*^	Mucin-7
	P02647	Apolipoprotein A-I
	Q9UPN3	Microtubule-actin cross-linking factor 1, isoforms 1/2/3/5

Biological functions not directly related to host protection or unknown (*n* = 29)	G3CIG0	MUC19 variant 12
	A0A0C4DGN4	Zymogen granule protein 16 homolog B
	A0A024R9Y3	HECT, UBA, and WWE domain containing 1, isoform CRA_a
	E7ETD6	Nucleosome-remodeling factor subunit BPTF
	P06733	alpha-Enolase
	A7E2D6	NAV2 protein
	H7BY35	Ryanodine receptor 2
	Q6P5S2	Protein LEG1 homolog
	F8WA11	CLIP-associating protein 1
	P02533	Keratin, type I cytoskeletal 14
	B2R825	alpha-1,4-Glucan phosphorylase
	B4DVQ0	cDNA FLJ58286, highly similar to actin, cytoplasmic 2
	Q0QET7	Glyceraldehyde-3-phosphate dehydrogenase (fragment)
	J3QLC9	Haptoglobin (fragment)
	B7Z565	cDNA FLJ54739, highly similar to alpha-actinin-1
	C0JYZ2	Titin
	C8C504	beta-Globin
	H6VRF8	Keratin 1
	P13645	Keratin, type I cytoskeletal 10
	B1APF8	cAMP-dependent protein kinase catalytic subunit beta (fragment)
	F6KPG5	Albumin (fragment)
	E9PKG6	Nucleobindin-2
	Q9Y6V0	Protein piccolo
	P10599	Thioredoxin
	O95661	GTP-binding protein Di-Ras3
	B4E1T1	cDNA FLJ54081, highly similar to keratin, type II cytoskeletal 5
	A0A087WWT3	Serum albumin
	A8K739	cDNA FLJ77339
	P35527	Keratin, type I cytoskeletal 9

^*∗*^Proteins involved in more than one process related to host protection.

## Supplementary Materials

Supplementary Materials Supplemental Table  1: detailed information regarding the identification of the 27 proteins that were common in all three used proteomic approaches.
