# Supplementary material for: Revealing the Amylase Interactome in Whole Saliva Using Proteomic Approaches
Source: Biomed Res Int. 2018 Jan 31;2018:6346954. doi: 10.1155/2018/6346954 (PMC5831883; doi:10.1155/2018/6346954)
Supplement: Supplementary Materials — Supplemental Table 1: detailed information regarding the identification of the 27 proteins that were common in all three used proteomic approaches. [file 6346954.f1.docx]

**Supplemental Table 1.** Detailed information regarding the identification of the 27 proteins that were common in all three used proteomic approaches.

| Accession Number | Protein name | Used proteomic approach | Number of replicates identified | Unique peptides (average) | Score (average) | % Coverage (average) |
| --- | --- | --- | --- | --- | --- | --- |
| C0JYZ2 | **Titin** | **In-solution** | **8** | **8.63** | **41.76** | **0.68** |
|  |  | **In-gel (SDS-PAGE)** | **6** | **N/A** | **N/A** | **N/A** |
|  |  | **In-gel (Native-PAGE)** | **5** | **6.8** | **113.36** | **0.38** |
| B4E1M1 | **cDNA FLJ60391, highly similar to Lactoperoxidase** | **In-solution** | **2** | **2** | **10.28** | **4.59** |
|  |  | **In-gel (SDS-PAGE)** | **4** | **N/A** | **N/A** | **N/A** |
|  |  | **In-gel (Native-PAGE)** | **4** | **4.5** | **32.26** | **8.12** |
| Q9HC84 | **Mucin-5B** | **In-solution** | **4** | **6** | **31.22** | **1.63** |
|  |  | **In-gel (SDS-PAGE)** | **9** | **N/A** | **N/A** | **N/A** |
|  |  | **In-gel (Native-PAGE)** | **3** | **3.67** | **14.59** | **1.9** |
| P04080 | **Cystatin-B** | **In-solution** | **8** | **2.38** | **19.83** | **32.53** |
|  |  | **In-gel (SDS-PAGE)** | **8** | **N/A** | **N/A** | **N/A** |
|  |  | **In-gel (Native-PAGE)** | **2** | **2.5** | **18.82** | **35.21** |
| B4DVQ0 | **cDNA FLJ58286, highly similar to Actin, cytoplasmic 2** | **In-solution** | **2** | **5** | **77.4** | **19.82** |
|  |  | **In-gel (SDS-PAGE)** | **9** | **N/A** | **N/A** | **N/A** |
|  |  | **In-gel (Native-PAGE)** | **4** | **4.75** | **46.9** | **27.7** |
| P01037 | **Cystatin-SN** | **In-solution** | **2** | **1.4** | **82.19** | **32.62** |
|  |  | **In-gel (SDS-PAGE)** | **8** | **N/A** | **N/A** | **N/A** |
|  |  | **In-gel (Native-PAGE)** | **4** | **2.5** | **46.67** | **32.62** |
| Q6PJF2 | **IGK@ protein** | **In-solution** | **2** | **5.5** | **57.87** | **33.41** |
|  |  | **In-gel (SDS-PAGE)** | **5** | **N/A** | **N/A** | **N/A** |
|  |  | **In-gel (Native-PAGE)** | **2** | **5** | **117.65** | **28.94** |
| Q0QET7 | **Glyceraldehyde-3-phosphate dehydrogenase (Fragment)** | **In-solution** | **2** | **2.5** | **11.46** | **17.18** |
|  |  | **In-gel (SDS-PAGE)** | **6** | **N/A** | **N/A** | **N/A** |
|  |  | **In-gel (Native-PAGE)** | **4** | **2** | **15.98** | **12.67** |
| A0A075B6K9 | **Ig lambda-2 chain C regions (Fragment)** | **In-solution** | **4** | **2.5** | **18.89** | **36.8** |
|  |  | **In-gel (SDS-PAGE)** | **4** | **N/A** | **N/A** | **N/A** |
|  |  | **In-gel (Native-PAGE)** | **4** | **2.75** | **27.83** | **37.03** |
| P05109 | **Protein S100-A8** | **In-solution** | **2** | **2** | **18.55** | **19.35** |
|  |  | **In-gel (SDS-PAGE)** | **9** | **N/A** | **N/A** | **N/A** |
|  |  | **In-gel (Native-PAGE)** | **2** | **2.5** | **17.82** | **24.73** |
| P12273 | **Prolactin-inducible protein** | **In-solution** | **9** | **4.56** | **54.77** | **31.05** |
|  |  | **In-gel (SDS-PAGE)** | **8** | **N/A** | **N/A** | **N/A** |
|  |  | **In-gel (Native-PAGE)** | **7** | **2.86** | **28.44** | **25.15** |
| Q96DR5 | **BPI fold-containing family A member 2** | **In-solution** | **8** | **6.13** | **42.84** | **21.79** |
|  |  | **In-gel (SDS-PAGE)** | **9** | **N/A** | **N/A** | **N/A** |
|  |  | **In-gel (Native-PAGE)** | **3** | **4.67** | **30.08** | **19.68** |
| A0A0C4DGN4 | **Zymogen granule protein 16 homolog B** | **In-solution** | **9** | **4.22** | **61.57** | **40.14** |
|  |  | **In-gel (SDS-PAGE)** | **8** | **N/A** | **N/A** | **N/A** |
|  |  | **In-gel (Native-PAGE)** | **6** | **2.83** | **28** | **22** |
| Q9UGM3 | **Deleted in malignant brain tumors 1 protein** | **In-solution** | **6** | **2.17** | **13.7** | **7.89** |
|  |  | **In-gel (SDS-PAGE)** | **9** | **N/A** | **N/A** | **N/A** |
|  |  | **In-gel (Native-PAGE)** | **6** | **2.83** | **19.83** | **9.39** |
| P01833 | **Polymeric immunoglobulin receptor** | **In-solution** | **9** | **3.33** | **27.07** | **6.81** |
|  |  | **In-gel (SDS-PAGE)** | **9** | **N/A** | **N/A** | **N/A** |
|  |  | **In-gel (Native-PAGE)** | **6** | **3.17** | **21.22** | **5.08** |
| P01876 | **Ig alpha-1 chain C region** | **In-solution** | **8** | **3.75** | **30.29** | **12.85** |
|  |  | **In-gel (SDS-PAGE)** | **7** | **N/A** | **N/A** | **N/A** |
|  |  | **In-gel (Native-PAGE)** | **5** | **3.6** | **24.01** | **15.33** |
| P23280 | **Carbonic anhydrase VI** | **In-solution** | **9** | **6** | **102.8** | **26.37** |
|  |  | **In-gel (SDS-PAGE)** | **9** | **N/A** | **N/A** | **N/A** |
|  |  | **In-gel (Native-PAGE)** | **8** | **3.88** | **72.81** | **14.65** |
| C8C504 | **Beta-globin** | **In-solution** | **3** | **2** | **12.69** | **17.01** |
|  |  | **In-gel (SDS-PAGE)** | **5** | **N/A** | **N/A** | **N/A** |
|  |  | **In-gel (Native-PAGE)** | **3** | **3** | **21.96** | **35.63** |
| A7Y9J9 | **Mucin 5AC, oligomeric mucus/gel-forming** | **In-solution** | **7** | **4.29** | **24** | **1.91** |
|  |  | **In-gel (SDS-PAGE)** | **2** | **N/A** | **N/A** | **N/A** |
|  |  | **In-gel (Native-PAGE)** | **4** | **5** | **30.91** | **1.62** |
| P01834 | **Ig kappa chain C region** | **In-solution** | **5** | **3.8** | **50.95** | **45.01** |
|  |  | **In-gel (SDS-PAGE)** | **6** | **N/A** | **N/A** | **N/A** |
|  |  | **In-gel (Native-PAGE)** | **4** | **3.5** | **45.66** | **49.06** |
| H6VRF8 | **Keratin 1** | **In-solution** | **7** | **7.29** | **67.35** | **13.73** |
|  |  | **In-gel (SDS-PAGE)** | **9** | **N/A** | **N/A** | **N/A** |
|  |  | **In-gel (Native-PAGE)** | **8** | **11.13** | **118.26** | **21.91** |
| P13645 | **Keratin, type I cytoskeletal 10** | **In-solution** | **4** | **6.25** | **47.49** | **13.44** |
|  |  | **In-gel (SDS-PAGE)** | **9** | **N/A** | **N/A** | **N/A** |
|  |  | **In-gel (Native-PAGE)** | **8** | **5.88** | **50.54** | **14.01** |
| P01036 | **Cystatin-S** | **In-solution** | **9** | **2.67** | **86.01** | **30.5** |
|  |  | **In-gel (SDS-PAGE)** | **8** | **N/A** | **N/A** | **N/A** |
|  |  | **In-gel (Native-PAGE)** | **5** | **1.8** | **35.47** | **20.57** |
| B2R4M6 | **Protein S100** | **In-solution** | **6** | **4.5** | **103.97** | **47.66** |
|  |  | **In-gel (SDS-PAGE)** | **9** | **N/A** | **N/A** | **N/A** |
|  |  | **In-gel (Native-PAGE)** | **8** | **3.88** | **52.34** | **43.86** |
| P35908 | **Keratin, type II cytoskeletal 2 epidermal** | **In-solution** | **2** | **4.5** | **41.9** | **12.6** |
|  |  | **In-gel (SDS-PAGE)** | **9** | **N/A** | **N/A** | **N/A** |
|  |  | **In-gel (Native-PAGE)** | **6** | **4.67** | **55.87** | **13.96** |

N/A (Not Applicable)– Refers to the samples from the In-gel (SDS-PAGE) approach where each gel lane was divided into 6 parts for individual digestion and MS/MS analysis. Averaging the number of unique peptides, score and % coverage for the samples submitted to this approach is not applicable since the relative protein abundance cannot be compared among the analyzed areas from the same lane.

Supplemental Table 1. Crosara et al.
